# Senescence‐associated β‐galactosidase reveals the abundance of senescent CD8+ T cells in aging humans

**DOI:** 10.1111/acel.13344

**Published:** 2021-05-03

**Authors:** Ricardo I. Martínez‐Zamudio, Hannah K. Dewald, Themistoklis Vasilopoulos, Lisa Gittens‐Williams, Patricia Fitzgerald‐Bocarsly, Utz Herbig

**Affiliations:** ^1^ Center for Cell Signaling Rutgers‐New Jersey Medical School Rutgers Biomedical and Health Sciences Rutgers University Newark New Jersey USA; ^2^ Department of Microbiology, Biochemistry, and Molecular Genetics Rutgers‐New Jersey Medical School Rutgers Biomedical and Health Sciences Rutgers University Newark New Jersey USA; ^3^ Rutgers School of Graduate Studies Rutgers‐New Jersey Medical School Rutgers Biomedical and Health Sciences Rutgers University Newark New Jersey USA; ^4^ Center for Immunity and Inflammation Rutgers‐New Jersey Medical School Rutgers Biomedical and Health Sciences Rutgers University Newark New Jersey USA; ^5^ Department of Pathology, Immunology, and Laboratory Medicine Rutgers‐New Jersey Medical School Rutgers Biomedical and Health Sciences Rutgers University Newark New Jersey USA; ^6^ Department of Obstetrics, Gynecology and Women's Health Rutgers‐New Jersey Medical School Rutgers Biomedical and Health Sciences Rutgers University Newark New Jersey USA

**Keywords:** aging, cellular senescence, immunosenescence, lymphocytes, p16, PBMC, senescence‐associated β‐galactosidase, T cells, telomere

## Abstract

Aging leads to a progressive functional decline of the immune system, rendering the elderly increasingly susceptible to disease and infection. The degree to which immune cell senescence contributes to this decline remains unclear, however, since markers that label immune cells with classical features of cellular senescence accurately and comprehensively have not been identified. Using a second‐generation fluorogenic substrate for β‐galactosidase and multi‐parameter flow cytometry, we demonstrate here that peripheral blood mononuclear cells (PBMCs) isolated from healthy humans increasingly display cells with high senescence‐associated β‐galactosidase (SA‐βGal) activity with advancing donor age. The greatest age‐associated increases were observed in CD8+ T‐cell populations, in which the fraction of cells with high SA‐βGal activity reached average levels of 64% in donors in their 60s. CD8+ T cells with high SA‐βGal activity, but not those with low SA‐βGal activity, were found to exhibit features of telomere dysfunction‐induced senescence and p16‐mediated senescence, were impaired in their ability to proliferate, developed in various T‐cell differentiation states, and had a gene expression signature consistent with the senescence state previously observed in human fibroblasts. Based on these results, we propose that senescent CD8+ T cells with classical features of cellular senescence accumulate to levels that are significantly higher than previously reported and additionally provide a simple yet robust method for the isolation and characterization of senescent CD8+ T cells with predictive potential for biological age.

## Graphical abstract

Senescent CD8+ T cells in peripheral blood can be detected, quantified, and isolated using a fluorogenic and self‐immobilizing substrate of senescence‐associated β‐galactosidase. Characterization of CD8+ T cells with high SA‐βGal activity isolated from healthy donors in their 20s and 60s revealed a significantly greater abundance of SA‐βGal expressing CD8+ T cells with a unique transcriptional signature and features telomere dysfunction‐induced senescence and p16‐mediated senescence in older humans.

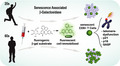

## 1 INTRODUCTION

Cellular senescence is a stable proliferative arrest that is encountered by mammalian cells in response to a variety of signals and stresses (Hernandez‐Segura et al., 2018). In mammals, this response functions to suppress cancer development and plays also important roles during tissue repair, wound healing, and embryonic development (Rhinn et al., 2019). Although senescent cells (SCs) can be cleared from tissue by adaptive and innate immune responses under these circumstances, not all SCs are cleared and, consequently, accumulate in various tissues during aging (Prata et al., 2019). In mouse models, this age‐associated accumulation of SCs has a substantial negative impact on fitness and health, as it contributes to aging and the development of age‐associated diseases (Baker et al., 2016; Munoz‐Espin & Serrano, 2014). While it is currently unclear why some SCs evade immune cell clearance and accumulate in tissues, it is possible that immune cells themselves progressively become senescent with age, thereby increasingly weakening immune responses that would otherwise clear SCs from aged tissue (Prata et al., 2019). This hypothesis, however, has been proven challenging to test primarily due to the lack of a universal marker that can identify all senescent mammalian immune cells accurately and efficiently (Goronzy & Weyand, 2019; Pangrazzi & Weinberger, 2020).

In mammalian cells, at the least two pathways activate the senescence program. One pathway, mediated by the tumor suppressor p53 and cyclin‐dependent kinase (CDK) inhibitor p21, primarily becomes activated in response to a persistent DNA damage response (DDR), such as the one caused due to telomere dysfunction. In normal somatic human cells, or cells that lack detectable telomerase activity, dysfunctional telomeres are generated not only due to repeated cell division cycles that cause progressive telomere erosion, but also as a consequence of genotoxic stresses that cause double‐stranded DNA breaks (DSBs) in telomeric repeats (Fumagalli et al., 2012; Hewitt et al., 2012). A second senescence pathway is activated due to upregulation of the CDK4/6 inhibitor p16^INK4a^, which results in a stable pRb‐dependent cell cycle arrest. While genotoxic stresses *can* activate this senescence pathway in certain cell types, p16^INK4a^ is also upregulated in the absence of telomere dysfunction or a persistent DDR (Ben‐Porath & Weinberg, 2005; Herbig et al., 2004). Although the molecular triggers of this DDR‐independent senescence response are still largely unclear, p16^INK4a^‐mediated senescence clearly has important physiological consequences, as it significantly contributes to aging and the development of age‐related disorders in mammals (Munoz‐Espin & Serrano, 2014).

Despite differences in pathway activation, SCs share a number of features. One characteristic that is common to all SCs is that they secrete numerous NF‐*k*B and p38‐MAPK regulated pro‐inflammatory cytokines and other molecules, collectively called the “senescence‐associated secretory phenotype” or SASP (Coppe et al., 2008). Although the SASP differs in composition depending on cell type, senescence‐inducing signals, and time elapsed following senescence induction, a primary function of the SASP is to generate a pro‐inflammatory environment that stimulates an immune response (Prata et al., 2019). Another feature common to SCs is that they up‐regulate certain heterochromatin proteins, such as macroH2A, leading to a stable repression of cell proliferation genes (Zhang et al., 2005, 2007). In addition, SCs develop a greater abundance of lysosomal content and a reduction in lysosomal pH (Kurz et al., 2000), resulting in increased expression and activity of lysosomal β‐galactosidase; this hallmark of SCs in particular allows their detection in cultures and in tissue, regardless of the senescence‐inducing signal or senescence pathway activated (Gorgoulis et al., 2019).

Our current understanding of cellular senescence stems primarily from studies conducted using mammalian fibroblast cultures. Senescence pathways in other cell types, including those of circulating peripheral blood mononuclear cells (PBMCs), such as CD4+ T cells, CD8+ T cells, monocytes, B cells, natural killer (NK) cells, and plasmacytoid dendritic cells (pDC's), are still incompletely understood (Vicente et al., 2016). Although subsets of PBMCs, such as cytotoxic CD8+ T cells, undergo replicative senescence in culture, to what degree they do so in vivo remains unclear (Chou & Effros, 2013; Pangrazzi & Weinberger, 2020). A primary reason for this uncertainty is that specific markers used to identify senescent CD8+ T cells (Song et al., 2018; Xu & Larbi, 2017), such as a loss of the cell surface receptors CD28 and CD27 and a gain of expression of CD45RA, CD57, TIGIT, and/or KLRG1 may not accurately characterize all T cells that have permanently lost the ability to proliferate due to acquisition of macromolecular damage, upregulation of cyclin‐dependent kinase (CDK) inhibitors, and development of senescence‐associated β‐Galactosidase (SA‐βGal) activity, criteria that define the state of cellular senescence (Gorgoulis et al., 2019). In fact, bulk CD8+ T cells that have lost expression of CD28 and/or that display varying levels of CD45RA, CD57, TIGIT, or KLRG1 maintain the ability to proliferate following appropriate stimulation, which is incompatible with a classical senescence response (Chou & Effros, 2013; Song et al., 2018).

Here, we report an accurate and efficient method to quantify, isolate, and characterize live senescent immune cells from peripheral blood of human donors. We reveal the identity of PBMC subsets that increasingly develop high levels of SA‐βGal activity in healthy humans with age, uncover potential causes for cellular senescence in circulating CD8+ T cells, and characterize the senescence pathways activated in CD8+ T cells with high SA‐βGal activity. Together, our findings may provide insights into therapeutic opportunities to modulate T‐cell senescence in disease, infection, and advanced age.

## 2 RESULTS

### 2.1 A method to isolate live SCs for subsequent analysis

One feature that is common to SCs is that they develop increased SA‐βGal activity (Gorgoulis et al., 2019). This hallmark of SCs allows their detection using chromogenic (X‐Gal) (Dimri et al., 1995) or fluorogenic (FDG) (Debacq‐Chainiaux et al., 2009) βGal substrates, albeit with limitations due to poor cell permeability or lack of cellular retention, respectively. To mitigate these limitations, we tested a second‐generation, cell‐permeable, and self‐immobilizing fluorogenic SA‐βGal substrate (fSA‐βGal) (Nakamura et al., 2017) for its ability to label live SCs for prolonged periods, so that they can be accurately analyzed, quantified, and isolated by flow cytometry. As anticipated, incubation with fSA‐βGal caused GM21 fibroblasts in oncogene‐induced senescence (OIS), either alone or mixed at a 3:1 ratio with non‐senescent GM21 fibroblasts, to develop significantly increased fluorescence signal intensities compared to proliferating non‐senescent control fibroblasts (Figure S1a). Live cells in OIS could be accurately sorted and isolated from non‐SCs by FACS, allowing us to conduct cell proliferation assays, immunofluorescence analysis, and gene expression profiling. We show that FACS‐isolated cells with high fSA‐βGal activity (fSA‐βGal high) were significantly impaired in their ability to proliferate, suppressed expression of cyclin A, and displayed increased expression levels of senescence genes *p21*, *p16^INK4a^*, *IL1β*, and *IL8* compared to cells with low activity (fSA‐βGal low; Figure S1b–e). Similar results were obtained when comparing etoposide‐treated fibroblasts undergoing DNA damage‐induced senescence with proliferating non‐senescent fibroblasts (Figures S1f–h). Collectively, these results demonstrate that fSA‐βGal is specific to labeling live human SCs and is efficient for isolating them from mixed cell populations for subsequent analysis.

### 2.2 Subsets of human PBMCs increasingly develop high SA‐βGal activity with advancing age

**FIGURE 1:**
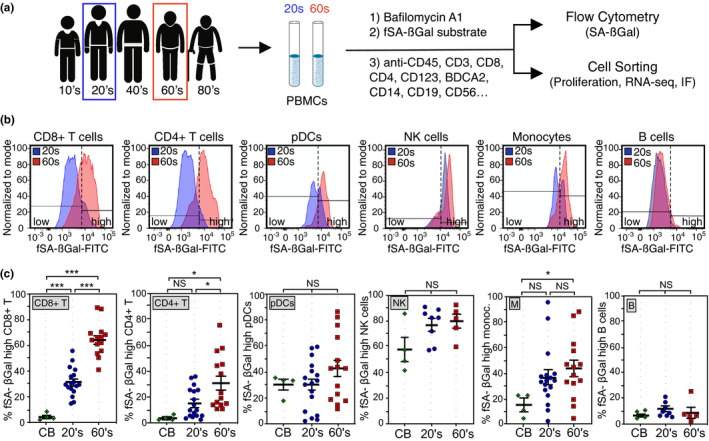
Humans display increased percentages of T lymphocytes with high fSA‐βGal signal intensities in advanced age. (a) Experimental strategy to quantify, isolate, and characterize senescent subsets of PBMCs from donors in their 20s and 60s. IF: immunofluorescence analysis. (b) Representative fSA‐ßGal intensity profiles and gates used to quantify fSA‐ßGal high cells for indicated PBMC subsets from a younger (20s, blue) and older (60s, red) donor. (c) Quantification of the percentages of fSA‐ßGal high cells in cord blood (CB), donors in their 20s (blue) and donors in their 60s (red) for indicated PBMC subsets. Whiskers indicate mean ± SEM and are indicated for each subset. Statistical significance was determined by an unpaired, two‐tailed Student's *t* test. ***p < 0.0001; **p = 0.0005; *p < 0.05; NS: not significant

To determine whether subsets of circulating human peripheral blood mononuclear cells (PBMCs) undergo cellular senescence as a function of donor age in circulation, we collected and analyzed blood from healthy human donors in two age groups: (a) “20s,” which included donors between the ages 23 and 30 years (average age 25 years; also referred to as “younger”), and (b) “60s” which included donors between the ages 57 and 67 years (average age 64 years; also referred to as “older”); around the age, the human immune system is found to exhibit age‐associated deficits (Table S1). Younger and older donors were always recruited in pairs, allowing us to process and analyze freshly isolated PBMCs from donor pairs in parallel (Figure 1a and Figure S2a). Cord blood, together with blood from healthy donors in their 20s, was also collected and analyzed for the presence of SCs, reasoning that if senescent cells increasingly develop with advancing age in humans, infant cord blood, essentially representing age 0, should display the fewest numbers of cells with high SA‐βGal activity. Surprisingly, age‐associated increases of mean fluorescence intensities (MFIs) of SA‐βGal signals were observed in most PBMC subsets analyzed for some donor pairs, including T lymphocytes, plasmacytoid dendritic cells (pDC's), natural killer (NK) cells, and monocytes (Figure 1b,c and Figure S2b). Most consistent age‐associated increases in mean fluorescence intensities (MFIs) of fSA‐βGal, however, were observed only for CD8+ T cells (Figure S2b,c). In order to determine which PBMC subsets increasingly develop high SA‐βGal activity with advancing age, we quantified the percentages of cells that display increased fSA‐βGal signal intensities by taking advantage of the fact that donors in their 20s, and often also older donors, had distinct populations of cells with lower and higher fSA‐βGal fluorescence. This allowed us to set gates at intersections where the two populations met, designating the population with lower signal intensity as “fSA‐βGal low” and the population with higher signal intensity as “fSA‐βGal high” (Figure 1b). Quantitative analysis of fSA‐βGal high cells revealed statistically significant age‐associated increases in human T lymphocytes, particularly in CD8+ T cells. For this subset, we discovered a striking age‐associated increase of cells with high SA‐βGal activity, ranging from 30 ± 3% in blood from donors in their 20s to 64 ± 4% in donors in their 60s, with some donors even displaying over 85% CD8+ T cells with high SA‐βGal activity (Figure 1c; *p* < 0.0001). CD4+ T cells also displayed a significant age‐associated increase of cells with high fSA‐βGal activity, albeit to a lesser degree compared to CD8+ T cells (15 ± 3% in younger and 31 ± 6% in older donors, *p* = 0.02). No sex differences in the percent of fSA‐βGal high cells or in MFIs of fSA‐βGal signal intensities were observed in either T‐cell population (Figure S2d). Not surprisingly, percentages of cells with high SA‐βGal activity were the lowest in cord blood, including in T lymphocytes, which contained fewer than 5% of fSA‐βGal high cells (Figure 1c and Figure S2d–f). Our data therefore demonstrate that an increasing proportion of T lymphocytes, in particular CD8+ T cells, develop high SA‐βGal activity with age.

### 2.3 CD8+ T cells with high SA‐βGal activity display features of telomere dysfunction‐induced senescence (TDIS) and p16^INK4a^‐mediated senescence

**FIGURE 2:**
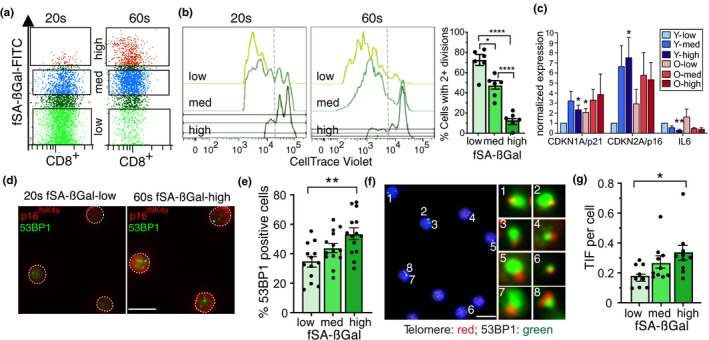
CD8+ T cells with high fSA‐βGal signal intensities are senescent. (a) Representative dot plot illustrating gates used to sort and isolate CD8+ T cells by FACS from a donor in their 20s and 60s, as indicated, based on low, inter(med)iate, and high fSA‐βGal signal intensities. (b) Representative Cell Trace Violet histograms of CD8+ T cells, sorted as in (a), following anti‐CD3 and anti‐CD28 stimulation for 5 days from a donor in their 20s and 60s as indicated. Bar graph, quantification of more than two cell divisions of CD8+ T cells from 6 healthy donors (3 donors in their 20s, 3 donors in their 60s) following anti‐CD3 and anti‐CD8 stimulation for 5 days. *p = 0.0002; **p < 0.0001. (c) RT‐qPCR expression profiles for indicated senescence‐associated genes in CD8+ T cells at indicated fSA‐ßGal levels in donors in their 20s (blue) and donors in their 60s (red). Average and SEM of five (CDKN1A and CDKN2A) and three (IL6) independent experiments. Statistical significance was determined by a two‐tailed unpaired t test, with Y‐low as the reference value. *p < 0.05; **p < 0.001; Y: younger donors in their 20s, O: Older donors in their 60s. (d) Immunofluorescence analysis of sorted CD8+ T cells from a donor in their 20s and 60s, as indicated, using antibodies against 53BP1 (green) and p16^INK4a^ (red). Outline of cell nuclei is indicated with white border. Scale bar: 10 μm. (e) Quantification of the percentage of CD8+ T cells positive for 53BP1 foci at each fSA‐ßGal level in younger (*n* = 8) and older donors (*n* = 8) combined. Whiskers depict mean ± SEM. Statistical significance was determined by a one‐way ANOVA. **p = 0.001. (f) Sorted CD8+ T cells with high fSA‐βGal signal intensities were simultaneously immunostained using antibodies against 53BP1 (green) and analyzed by FISH to detect telomeres (red). Blue: DAPI. Enlarged versions of the numbered DNA damage foci showing co‐localization with telomeres are shown in the right micrographs. Scale bar: 10 μm. (g) Quantification of mean TIF per cell in sorted CD8+ T cells, as indicated, in donors in their 20s (*n* = 5) and 60s (*n* = 5) combined. Whiskers depict mean ± SEM. Statistical significance was calculated by a one‐way ANOVA. *p = 0.0280

To test whether CD8+T cells with high SA‐βGal activity display other characteristics of senescent cells, we sorted and collected them by FACS based on low, intermediate, or high fSA‐βGal signal intensities (Figure 2a and Figure S3a) and measured cell proliferation, senescence gene expression, and development of features specific to SCs. Significantly, the ability of CD8+ T cells to proliferate following activation was inversely proportional to fSA‐βGal signal intensities. While 73 ± 5% of cells with low SA‐βGal activity were able to undergo more than two cell divisions during a 5‐day period of stimulation with anti‐CD3 and anti‐CD‐28, the fractions of proliferating cells with intermediate and high fSA‐βGal signal intensities were reduced to 48% ± 4% and 12 ± 3%, respectively, under the same conditions (Figure 2b). No significant differences in proliferation capacities were observed between T cells collected from donors in their 20s and 60s at respective fSA‐βGal signal intensities (Figure S3b). Gene expression analysis by RT‐qPCR demonstrated increased expression levels of senescence genes p16^INK4a^ and p21 in cells with high SA‐βGal activity compared to cells with intermediate or low activities (Figure 2c), which is consistent with a senescence response. Levels of IL6 mRNA, however, were decreased in cells with high SA‐βGal activity, which is unlike senescent human fibroblasts that develop high IL6 expression levels (Coppe et al., 2008). In addition, immunofluorescence analysis revealed that protein levels of p16^INK4a^ and the senescence marker macroH2A were the lowest in CD8+ T cells with low SA‐βGal activity and increased proportionally in cells with intermediate and high SA‐βGal activities (Figure 2d and Figure S3c,d). Similarly, cells that displayed multiple foci of 53BP1, a DDR factor that localizes to sites of DSBs, were less frequently detected in cells with low SA‐βGal activity compared to cells with intermediate or high activities, irrespective of donor age (Figure 2d,e and Figure S3e–g). Approximately 40% of DDR foci analyzed were localized at telomeric repeats, revealing that a substantial fraction of DSBs in CD8+ T cells are caused as a result of telomere dysfunction (Figure 2f and Figure S3h). Overall, the mean number of telomere dysfunction‐induced DNA damage foci (TIF) per cell increased proportionally with increasing SA‐βGal activity, irrespective of donor age, demonstrating a direct correlation between SA‐βGal activity and presence of dysfunctional telomeres in CD8+ T cells (Figure 2g and Figure S3h,i). Our data therefore demonstrate that humans increasingly develop CD8+ T cells with features of TDIS and p16^INK4a^‐mediated senescence with advancing age.

### 2.4 SA‐βGal expressing CD8+ T cells develop a senescence gene expression signature

**FIGURE 3:**
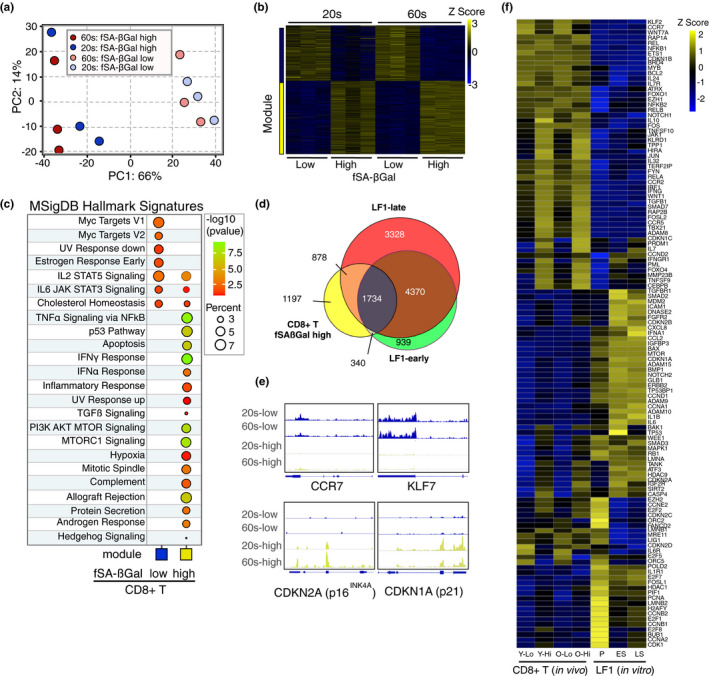
CD8+ T cells with high fSA‐βGal signal intensities develop a gene expression signature that is characteristic of senescent human cells. (a) Principal component analysis on transcriptomes from SA‐βGal high and low CD8 cells isolated from 3 donors in their 20s and 3 donors in their 60s. (b) Expression heatmap of the DEGs within each module across fSA‐ßGal low and high CD8+ T cells. Each column represents an individual donor. Data are represented as Z‐scores. (c) Functional over‐representation map depicting Molecular Signatures Database (MSigDB) hallmark gene sets associated to each transcriptomic cluster. Circles are color‐coded according to the FDR‐corrected *p*‐value based on the hypergeometric distribution test. (d) Venn diagram portraying the intersections and disjunctive unions of differentially expressed genes in CD8+ T cells with high fSA‐ßGal activity (yellow) and human lung fibroblasts (LF1) that had remained in replicative senescence for 2 months (LF1‐early; green) and 4 months (LF1‐late; red). (e) Representative genome browser visualizations of normalized reads at indicated gene loci in CD8+ T cells sorted based on low and high fSA‐ßGal signal intensities from a representative donor in their 20s and 60s, as indicated, using RNA‐seq. (f) Expression heatmap of a selection of senescence‐associated genes in CD8+ T cells isolated from peripheral blood and sorted based on low (Lo) and high (Hi) fSA‐ßGal signal intensities from younger (Y; 20s) and older (O, 60s) donors and in vitro cultured LF1 fibroblasts in proliferation (P), early (ES; 2 months) and late senescence (LS; 4 months) as in (d). Each column represents the average expression of 3 independent donors (CD8+) and 3 independent experiments (LF1)

To characterize the phenotype of CD8+ T cells with these hallmark features of cellular senescence in greater detail, we performed RNA‐sequencing on CD8+ T cells with high SA‐βGal signal intensities, sorted as in Figure 2a, from 3 healthy human donors in their 20s and 3 healthy human donors in their 60s. As a control, RNA from CD8+ T cells with low SA‐βGal signal intensities from the same donors was also sequenced. Significantly, principal component analysis (PCA) and hierarchical clustering revealed that the variability between the 12 transcriptomes was largely determined by SA‐βGal activity, not by donor age (Figure 3a and Figure S4a). A total of 4149 genes were differentially expressed between CD8+ T cells with high and low SA‐βGal activity, corresponding to 2362 upregulated and 1787 down‐regulated genes with a minimal fold change of 1.2. Weighted correlation network analysis (WGCNA) (Langfelder & Horvath, 2008) generated two gene modules, yellow and blue, displaying high co‐expression interconnectivity (Figure S4b–d). Consistent with the PCA, differentially expressed genes within each module were partitioned based on whether cells displayed high or low SA‐βGal activity and not based on donor age (Figure 3b). Functional over‐representation analysis enriched for a highly diverse array of biological processes consistent with a senescence response in the yellow module, including “p53 pathway,” “inflammatory response,” and “TGFβ signaling,” many of which overlapped with pathways also activated in senescent human fibroblasts (De Cecco et al., 2019; Martínez‐Zamudio et al., 2020) (Figure S4e,f). In contrast, the blue module was overall less diverse, enriching for pathways that included “IL2‐STAT5 signaling,” “IL6‐JAK‐STAT3 signaling,” and “MYC targets,” which are consistent with cell proliferation and normal CD8+ T‐cell function (Ross & Cantrell, 2018) (Figure 3c). Significantly, 71% of the 4149 differentially expressed genes (DEGs) of CD8+ T cells with high SA‐βGal activity were also differentially expressed in replicative senescent human fibroblasts, demonstrating a striking overlap in senescent gene regulation between the two cell types (Figure 3d). Interestingly, the gene expression pattern of CD8+ T cells with high SA‐βGal activity resembled a state of prolonged or deep senescence, as DEGs and pathways activated in these cells displayed a greater overlap with fibroblasts that had undergone replicative senescence for extended periods (4 months), rather than for a shorter period (2 months; Figure 3d and Figure S4f) (De Cecco et al., 2019).

A more detailed comparison of the expression of a subset of senescence‐associated genes revealed commonalities and cell type‐specific senescence gene expression profiles. Classical senescence‐associated genes, such as products of the *CDKN1* and *CDKN2* loci, were expressed in both senescent human fibroblasts and CD8+ T cells with high SA‐βGal activity, albeit at different magnitudes (Figure 3e,f). Expression of SASP genes, however, was more cell type‐specific. For instance, interleukins *(IL*)*1A*, *1B*, and *6*, were highly expressed in senescent fibroblasts but not in CD8+ T cells with high SA‐βGal activity. In contrast, expression levels of *TNF*, *TGFß1*, and *IFNG* were high in CD8+ T cells with high SA‐βGal activity, but not in senescent fibroblasts (Figure S4g). Overall, our data therefore demonstrate that human CD8+ T cells with high SA‐βGal activity develop a unique senescence gene expression signature in vivo, yet this signature overlaps substantially with that of replicative senescent human fibroblasts.

### 2.5 CD8+ T cells with high SA‐βGal activity increasingly develop with advancing age, irrespective of their differentiation state

**FIGURE 4:**
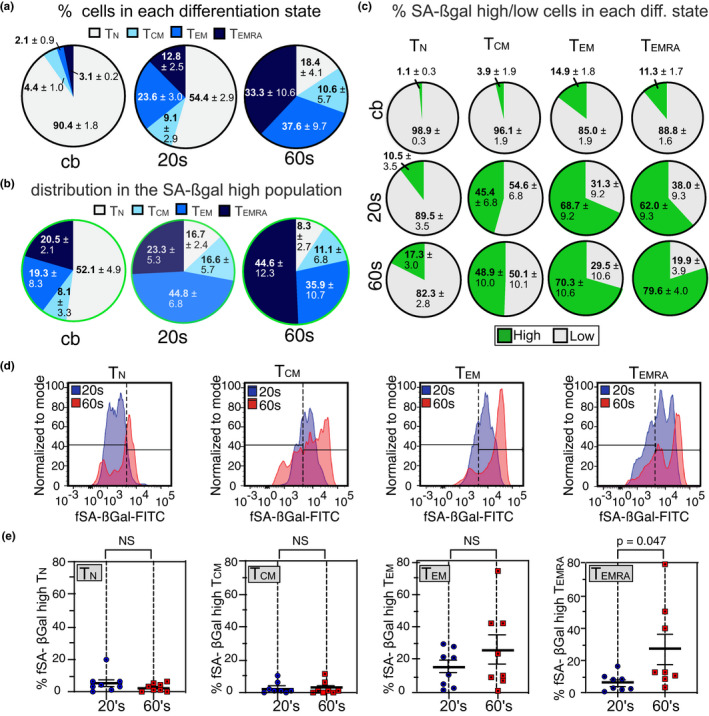
Senescent CD8+ T cells develop primarily in TEM and TEMRA subsets. (a) Distribution (% ± SEM) of indicated T‐cell differentiation states from cord blood (cb; *n* = 4), young (*n* = 8) and old (*n* = 8) donors. (b) Distribution (% ± SEM) of indicated T‐cell differentiation states from cord blood (cb; *n* = 4), donors in their 20s (*n* = 8) and donors in their 60s (*n* = 8) in fSA‐βGal high‐sorted CD8+ T‐cell populations. (c) Fraction of fSA‐βGal low (gray) and fSA‐βGal high (green) cells within indicated CD8+ T‐cell differentiation states from cord blood (top row), donors in their 20s (middle row) and donors in their 60s (bottom row). (d) Representative fSA‐ßGal intensity profiles and gates used to quantify fSA‐ßGal high cells for indicated CD8+ T‐cell differentiation states from a donor in their 20s (blue) and donors in their 60s (red). Gates were set based on the profiles generated by the total CD8+ T‐cell population (e) Quantification of the percentages of fSA‐ßGal high cells in donors in their 20s and 60s for indicated CD8+ T‐cell differentiation states. Statistical significance was determined by an unpaired, two‐tailed Student's *t* test. NS: not significant

CD8+ T‐cell populations can be divided into distinct subsets based on their functions, and the expression of cell surface receptors CCR7 and CD45RA, among others (Appay et al., 2008; Farber et al., 2014; Sallusto et al., 1999). Subsets include naïve (T_N_: CCR7+/CD45RA+), central memory (T_CM_: CCR7+/CD45RA‐), effector memory (T_EM_: CCR7‐/CD45RA‐), and effector (T_EMRA_: CCR7‐/CD45RA+) T cells. Although T_EMRA_ cells have been shown to display some features of cellular senescence, such as increased DNA damage, as well as reduced proliferative capacity, telomere lengths, and telomerase activity (Callender et al., 2018; Henson et al., 2014), it remains unclear whether they can be defined as senescent in the classical sense, particularly as T_EMRA_ cells retain function and their growth arrest is reversible (Goronzy & Weyand, 2019; Xu & Larbi, 2017). In line with previous studies (Song et al., 2018), we observed that the fraction of CD8+ T_EM_, and T_EMRA_ cells were significantly more abundant in donors in their 60s compared to younger donors or cord blood, while the fraction of T_N_ cells was dramatically decreased with age (Figure 4a and Figure S5a). Surprisingly, cells with high SA‐βGal activity were detected in all differentiation states, regardless of donor age (Figure 4b). While donors in their 20s developed CD8+ T cells with high SA‐βGal activity primarily in T_EM_ subsets, donors in their 60s displayed the greatest fraction of these cells in both the T_EM_ and T_EMRA_ subsets (Figure S4b,c). However, a significant percentage of T_EMRA_ cells (89%, 38%, and 20% in cord blood, donors in their 20s, and donors in their 60s, respectively) did not display increased SA‐βGal activity, demonstrating that T_EMRA_ cells are comprised of both fSA‐βGal high and fSA‐βGal low cells in all age groups analyzed (Figure 4c). Importantly, a fraction of T_N_ cells also displayed high SA‐βGal activity, particularly in donors in their 60s where 17.3 ± 3% T_N_ cells were found in the fSA‐βGal high population. Our data therefore demonstrate that CD8+ T cells with features of cellular senescence develop in all differentiation states, including in naïve T cells, which is consistent with previous reports (Figure 4b,c) (Pereira et al., 2020; Pulko et al., 2016; Quinn et al., 2018).

### 2.6 CD8+ T cells with high SA‐βGal activity are distinct from PD1+, CD57+, KLRG1+ CD8+ T cells, and from T_EMRA_ cells

**FIGURE 5:**
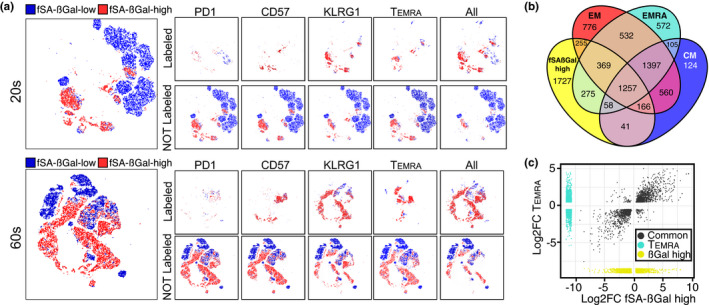
CD8+ T cells with high fSA‐ßGal signal intensities are phenotypically distinct from exhausted and senescent‐like cells and transcriptionally distinct from T_EMRA_ cells. (a) t‐SNE projections of CD8+ T cells with low (blue) and high (red) fSA‐ßGal signal intensities that were also labeled with indicated cell surface receptors. Gray: CD8+ T cells that immunostained for indicated cell surface antigens but that did not fall into the fSA‐ßGal low or high populations. (b) Venn diagrams portraying the intersections and disjunctive unions of DEGs in fSA‐ßGal high, T_CM_, T_EM_, and T_EMRA_ CD8+ T cells. (c) Correlation plot of the log2 fold changes of the DEGs in fSA‐ßGal high and EMRA CD8+ T cells. Dark gray points represent genes expressed in both populations. Turquoise dots are EMRA‐specific genes. Yellow dots are fSA‐ßGal high‐specific genes

T‐cell exhaustion is a phenotype caused by repeated antigenic stimulation by pathogens, resulting in functional impairment and the inability to produce cytokines such as IL‐2, TNFα, and IFNγ (Chou & Effros, 2013). Similar to T_EMRA_ cells, exhausted CD8+ T cells display characteristics of SCs such as shortened telomeres, absence of telomerase activity, and reduced proliferative capacity (Bellon & Nicot, 2017). In addition, markers such as KLRG1 and CD57 have been shown to be expressed on senescent‐like CD8+ T cells, yet whether any of these markers also labels CD8+ T cells with high SA‐βGal activity remains unclear (Chou & Effros, 2013; Xu & Larbi, 2017). To resolve this issue, we conducted t‐distributed Stochastic Neighbor Embedding (t‐SNE) analysis on CD8+ T cells from younger and older donors that were simultaneously treated with the fSA‐βGal substrate and immunostained for T‐cell exhaustion markers. This analysis revealed that only a small fraction of CD8+ T cells with high SA‐βGal activity expressed the inhibitory receptor PD1, a key feature of exhausted T cells (Figure 5a). In fact, PD1 was expressed to a large degree also on CD8+ T cell with low fSA‐βGal signal intensities, demonstrating that T‐cell exhaustion does not result in upregulation of SA‐βGal activity. Similarly, none of the other markers that have previously been reported to label senescent‐like CD8+ T cells, including CD57, KLRG1, or CCR7‐/CD45RA+ (T_EMRA_), were expressed exclusively on CD8+ T cells with high fSA‐βGal signal intensities, nor did they label all cells that displayed high levels of fSA‐βGal signal intensities (Figure 5a). Furthermore, T_EMRA_ cells, generally regarded as a T‐cell differentiation stage that most closely resembles a state of cellular senescence, displayed a gene expression signature that was markedly distinct to that from CD8+ T cells with high fSA‐βGal activity, as only 53% of DEGs were shared between these two cell states (Figure 5b,c and Figure S5). In fact, CD8+ T cells with high fSA‐βGal activity have a transcriptome that is substantially different from any of the known CD8+ T‐cell differentiation states (Figure S5), supporting our conclusion that human CD8+ T cells with high fSA‐βGal activity in peripheral blood represent a novel and unique T‐cell fate.

## 3 DISCUSSION

Cellular senescence is a state in which a cell permanently loses the ability to proliferate, undergoes dynamic intracellular changes that stabilize the proliferative arrest, and develops pronounced secretory activity to signal and influence its surrounding environment (Hernandez‐Segura et al., 2018). CD8+ T cells that display features consistent with a senescence response have been shown to be functionally impaired and more abundant in older humans, suggesting that they contribute to the decline of immune cell function in mammals (Callender et al., 2018; Henson et al., 2014; Quinn et al., 2018; Song et al., 2018). However, given that CD8+ T cells, previously characterized as senescent‐like based on expression of certain cell surface markers, regain function and the ability to proliferate under certain conditions, it remains possible that T‐cell senescence, and potentially also senescence of other PBMC subsets, is reversible and different compared to cellular senescence of other cell types. More likely, however, is that certain cell surface markers do not accurately distinguish all senescent from non‐senescent T cells that develop in vivo, which is supported by our data. Our observations that CD8+ T cells in vivo display defining features of cellular senescence, such as high levels of SA‐βGal activity, p16^INK4a^, macroH2A, dysfunctional telomeres, and a transcriptional signature that overlaps 71% with that of human fibroblasts that underwent replicative senescence in vitro, support our conclusion that a classical senescence response of CD8+ T cells exists. Given that the transcriptome of SA‐βGal expressing CD8+T cells in peripheral blood was more similar to that of human fibroblasts that were in a state of replicative senescence for 4 months, compared to that of fibroblasts that remained in senescence for only 2 months, our data also raise the possibility that that CD8+ T cells with high SA‐βGal activity persist in circulation for months or longer.

Although we also observed age‐associated increases in the percentages of cells with high fSA‐βGal signal intensities in most other subsets of human PBMCs, we currently cannot draw any conclusions whether cells in these subsets are also in a state of cellular senescence. A large proportion of NK cells, for example, displayed high levels of fSA‐βGal signal intensities regardless of donor age, which might be a consequence of their large content of lysosome‐related secretory organelles, rather than all of these cells being in a state of cellular senescence. Our conclusion that CD8+ T cell with high fSA‐βGal signal intensities are in a state of cellular senescence and increase in abundance with age, however, is consistent with previous reports. For example, transcript levels of the senescence marker p16^INK4a^ have been demonstrated to increase in T cells from healthy human donors in an age‐dependent manner (Liu et al., 2009). Similarly, senescent‐like CD8+ T_EMRA_, CD57, or KLRG1 expressing lymphocytes also increasingly develop with age in humans (Callender et al., 2020; Merino et al., 1998; Ouyang et al., 2003; Saule et al., 2006), albeit at significantly lower levels compared to the total SA‐βGal expressing CD8+ T population. Indeed, while most CD8+ T_EMRA_, CD57, or KLRG1 expressing cells are comprised largely, but not exclusively, of cells expressing high levels of SA‐βGal activity, our study additionally revealed that a significant proportion of SA‐βGal expressing cells also develop outside of these populations in an age‐associated manner. These observations suggest that previous studies potentially underestimated the fraction of senescent CD8+ T cells that develop in the periphery with advancing age.

The decline of immune function with age has been attributed to a number of factors, including to the age‐associated impairment in thymopoiesis, a reduction in the ratio of naïve to memory cells, and an increasing chronic low‐grade inflammation. These changes are hallmarks of “immunosenescence” and are considered to be the primary cause of the increased frequency and severity of disease and infections in the elderly. Surprisingly, impaired immune function can also accelerate the aging process, as it promotes the accumulation of senescent cells in a mouse model with defects in NK and T‐cell function and causes these animals to develop age‐associated disorders and shortened lifespan (Ovadya et al., 2018). Further supporting the conclusion that impaired T‐cell function promotes aging in mammals is a recent study demonstrating that mice with defective CD8+/CD4+ T cells, due to T‐cell‐specific knockout of the mitochondrial transcription factor A (Tfam), accumulated senescent cells in various tissues in an accelerated manner, which caused these mice to develop numerous age‐associated disorders and dramatically shortened their lifespan (Desdin‐Mico et al., 2020). Significantly, Tfam‐deficient T cells displayed key features of cellular senescence, such as increased lysosomal content, reduced lysosomal pH, deficiency in cell proliferation, a reduced NAD+/NADH ratio, and increased production of pro‐inflammatory cytokines TNFα and IFNγ, among others (Baixauli et al., 2015). The systemic senescence‐inducing and pro‐aging effects of these defective T cells were partially caused by their secreted molecules, as blocking TNFα suppressed development of senescent cells in various tissues and rescued many of the age‐associated disorders (Desdin‐Mico et al., 2020). Thus, maintaining functional T cells throughout life appears to be critically important, not only because T cells target and eliminate senescent cells that increasingly develop in aging tissues (Ovadya et al., 2018; Pereira et al., 2019), but also because defects in T‐cell function that lead them to develop senescence‐like features and increased production of pro‐inflammatory cytokines such as TNFα, exacerbates the systemic senescent cell burden and promotes aging in mammals (Desdin‐Mico et al., 2020).

Remarkably, most of the reported characteristics of Tfam‐deficient mouse T cells, including substantially increased expression of inflammatory cytokines TNFα, and IFNγ are shared also with senescent human CD8+ T cells, as our study reveals. The observations that up to 89% of CD8+ T cells display features of cellular senescence in healthy subjects in their 60s therefore raises the possibility that T‐cell senescence may be a major contributing factor to immunosenescence with potentially profound physiological consequences for humans. A large abundance of CD8+ T cells in peripheral blood of aged humans might therefore not only contribute to the increased susceptibility of the elderly to infections, but it might also impair clearance of senescent cells that accumulate in various tissues with age, accelerate development of systemic senescence through the active secretion of inflammatory SASP components, and thereby promote the development of age‐associated pathologies.

Our observations that telomeres are involved in senescence of CD8+ T cells opens opportunities to suppress immune cell senescence and improve immune surveillance in advanced age. This could be accomplished, for example, by enhancing telomerase activity or by reducing formation of dysfunctional telomeres using pharmacological means. Furthermore, compounds that neutralize the activity of pro‐inflammatory cytokines produced by senescent human CD8+ T cells such as TNFα, that are already proven to be effective in suppressing systemic senescence caused by senescent‐like T cells in mice (Desdin‐Mico et al., 2020), might also prove effective in suppressing the potentially damaging effects of senescent T cells in humans. Similarly, senolytics, drugs that target and eliminate senescent cells in tissue might also prove effective in eliminating senescent T cells in peripheral blood, although it remains to be determined whether this would improve immune function or be harmful due to elimination of cells that retain some functionality in senescence. Strategies to eliminate the damaging effects of senescent CD8+ T cells could potentially suppress age‐associated diseases and enhance immune responses toward infections. In addition, the ability to efficiently detect, quantify, isolate, and analyze senescent CD8+ T cells from human donor blood based on SA‐βGal activity may prove useful for diagnostic purposes, to serve not only as a biomarker of biological age, but also as a potential marker of acute and chronic diseases.

## 4 EXPERIMENTAL PROCEDURES:

### 4.1 Cell culture

GM21 fibroblasts were obtained from the Coriell Institute and cultured in Dulbecco's Modified Eagle's medium (DMEM) containing 10% FBS and 1X penicillin/streptomycin at 37˚C and 3% oxygen. GM21‐ER:RAS were generated by retroviral transduction as previously described (Puvvula et al., 2014). OIS was induced by addition of 200 nM 4‐hydroxytamoxifen (4‐OHT) for 14 days, with media and 4‐OHT being replenished every 3 days. For DNA damage/etoposide‐induced senescence, GM21 fibroblasts were treated with a single dose of 20 µM etoposide followed by incubation for 16 days. For both senescence models, SA‐ßGal activity was detected using the Cellular Senescence Detection Kit (Dojindo Molecular Technologies, Gaithersburg, MD), according to manufacturer's instructions. Briefly, control and senescent cells were collected by trypsinization, washed 2X in complete DMEM, and resuspended at a concentration of 1 × 10^6^ cells/ml. Cell suspensions (1 ml total volume) were incubated with 1X bafilomycin A1 solution for 1 h at 37°C. Subsequently, cells were incubated with 1X SPiDER‐ßGal for 30 min at 37°C. Cells were centrifuged at 2000 rpm for 4 min at RT, washed twice in PBS, and collected by an additional centrifugation step as described and analyzed as described in the main text.

### 4.2 EdU incorporation

Proliferating and senescent GM21‐ER:RAS fibroblasts as well as FACS‐sorted fSA‐ßGal low and high fibroblasts from mixed populations were seeded on collagen coated coverslips (Corning, Corning, NY) in 6‐well plates, supplemented with DMEM 10% FBS and antibiotics (1X penicillin/streptomycin). EdU incorporation was performed using the Click‐iT EdU Alexa Fluor Imaging Kit as per the manufacturer's instructions (Thermo Fisher Scientific, Waltham, MA). Cells were imaged using Images were acquired with a Zeiss Axio Observer Z1 Inverted Phase Contrast Fluorescence Microscope using a 10× objective and images analyzed using the with Zeiss ZEN 2.5 (blue edition) software.

### 4.3 Peripheral blood mononuclear cell isolation

The Institutional Review Board of New Jersey Medical School approved this study (ID Pro0119980237, approved November 14, 2019). Blood was obtained from healthy, consenting donors 23–65 years old in heparinized collection tubes. Details of human subjects analyzed are listed in Table S1. Peripheral blood mononuclear cells were separated using Lymphocyte Separation Medium (Corning, Manassas, VA) via density centrifugation following the manufacturer's protocol. PBMCs were cultured in RPMI 1640 (VWR Life Science, Radnor, PA) with l‐glutamine (Corning, Manassas, VA) supplemented with 10% heat‐inactivated fetal bovine serum (Gibco, Gaithersburg, MD), 100 U/ml penicillin, 100 mg/ml streptomycin, 100 mg/ml gentamicin, and 5 mM HEPES (Sigma Aldrich, St. Louis, MO). Cells were counted using the Cellometer Auto 2000 (Nexcelom, Lawrence, MA) and cultured at 2 × 10^6^ cells/ml.

### 4.4 Antibodies

APC anti‐CD54RA (HI100), PE Cy7 anti‐CCR7 (G043H7), PerCP Cy5.5 anti‐CD8(SK1), PerCP Cy5.5 anti‐CD8 (SK1), AF700 anti‐CD3 (HIT3A), APC anti‐CD3 (HIT3A), PE Cy7 anti‐CD4 (OKT4), PerCP Cy5.5 anti‐CD303 (210A), APC Cy7 anti‐CD14 (HDC14), BUV395 anti‐CD45 (H130), Brilliant Violet 510 anti‐CD57 (QA17A04), Brilliant Violet 711 anti‐PD1 (EH12.2H7), PE anti‐KLRG1 (14C2A07), Pacific Blue anti‐CD56 (5.1H11), AF700 anti‐CD19 (HIB19) were purchased from BioLegend (San Diego, CA), anti‐p16 (JC8, GeneTex, San Antonio, TX), anti‐53BP1 (polyclonal; Novus, Littleton, CO), anti‐macroH2A (H‐39, Santa Cruz, Dallas, TX). Secondary antibodies used for immunofluorescence staining were as follows: Cy3 donkey anti‐mouse (Jackson ImmunoResearch, West Grove, PE) and Alexa Fluor 488 goat anti‐rabbit (Invitrogen, Waltham, MA).

### 4.5 Surface staining

Using the Cellular Senescence Detection Kit‐SPiDER‐βGal (Dojindo Molecular Technologies, Inc, Rockville, MD), bafilomycin A1 was reconstituted in 30 μl of dimethyl sulfoxide (Sigma Aldrich, St. Louis, MO) and SPiDER‐βGal was reconstituted in 20 μl dimethyl sulfoxide. PBMCs were then incubated with a 1:500 dilution of bafilomycin A‐1 for 1.5 h before the addition of 1:500 dilution SPiDER‐βGal for another 30 min. PBMCs were washed with PBS (Corning, Manassas, VA) and incubated with 5 µl of heat‐inactivated purified human serum, collected from healthy donors, at room temperature for 5 min. Cells were then incubated for 20 min with antibody at 4 degrees and washed with 2% FCS (Gibco, Gaithersburg, MD), in PBS. To test for viability before cell sorting, cells were resuspended in 25 μl/ml 4′,6‐diamidino‐2‐phenylindole (DAPI (BioLegend, San Diego, CA) prior to sample collection. Samples for flow cytometric analysis were acquired at 300,000 events using an BD LSRFortessa X‐20 (BD Biosciences, Franklin Lakes, NJ), and analysis was performed with FlowJo software (FlowJo, (versions 9.3.2, 10) LLC Ashland, OR).

### 4.6 CD8+ T‐Cell enrichment

CD8+ T cells were isolated from PBMCs prepared as described, by negative selection using the StemCell EasySep Human CD8+ T‐Cell Isolation Kit (Cambridge, MA) according to the manufacturer's protocol. Purity of enriched cells was assessed via flow cytometry as the proportion of CD3+ and CD8+ cells with purities ranging from 95%–98%. CD8+ T cells were counted using the Cellometer Auto 2000 (Nexcelom, Lawrence, MA) and cultured at 2 × 10^6^ cells/ml. Cells were sorted based on fSA‐ßGal intensity on the BD FACSAria Fusion (BD Biosciences, Franklin Lakes, NJ).

### 4.7 CD8+ T‐Cell proliferation

50,000 sorted CD8+ T cells were stained with CellTrace Violet (ThermoFisher, Waltham, MA) following manufacturer's protocol. Labeled cells were mixed with 400,000 unlabeled autologous PBMCs and cultured in 200 µl media with 2 µg/ml plate‐bound anti‐CD3 (OKT3) and 5 µg/ml soluble anti‐CD28 (CD28.2) purchased from BioLegend (San Diego, CA). After 5 days, cells were collected and stained with Zombie UV Fixable Viability Kit (Biolegend, San Diego, CA), anti‐CD3, and anti‐CD8. Samples were acquired on a X‐20 BD LSRFortessa (BD Biosciences, Franklin Lakes, NJ). Sorted cells were identified as all CD3+ CD8+ CellTrace Violet+cells.

### 4.8 Senescence gene expression profiling

RNA from GM21 and CD8+ T‐cell populations described in this study was purified using a Macherey‐Nagel RNA XS Plus Kit according to the manufacturer's instructions (Macherey‐Nagel, Duren, Germany). cDNA was generated with a Bio‐Rad iScript cDNA synthesis kit according to the manufacturer's instructions (Bio‐Rad, Hercules, CA). For all genes assessed, Qiagen QuantiTect primer assays were used and real‐time RT‐qPCR was performed on a Bio‐Rad CFX96 Real‐Time PCR detection system (Bio‐Rad, Hercules, CA). Data were analyzed on the Bio‐Rad CFX Manager software 3.1.

### 4.9 Statistical analysis

With the exception of microarray and RNA‐seq analyses, all statistical tests utilized in this study were performed in GraphPad Prism 8 with the methodology as indicated in the legend of their respective figure.

### 4.10 Preparation of T cells for immunofluorescence staining

Immunofluorescence staining of T cells was performed as described (Tsang et al., 2017). Briefly, sorted CD8+ were collected and resuspended in PBS at 10^6^ cells/ml. The cell suspension was layered on glass coverslips and was left standing for 30 min to let the cells attach on the coverslip through gravity sedimentation. After 30 min, the cells were fixed in 4% formaldehyde for 15 min and permeabilized for 15 min with 0.2% Triton X PBS (PBST 0.2%). Following permeabilization, cells were incubated in blocking buffer (4% BSA in PBST 0.1%) for 1 h. Of note, the fixation procedure essentially eliminates fluorescence emitted by fSA‐βGal.

### 4.11 Immunofluorescence staining (IF)

Cells were incubated with primary antibodies diluted in blocking buffer for 2 h at room temperature and were subsequently washed 3 times (7 min each) with PBST 0.1%. Incubation with secondary antibodies was performed for 1 h at room temperature followed by 3 washes (7 min each) with PBS. Cells were then air‐dried and coverslips mounted on slides using DAPI containing mounting medium (Invitrogen, Waltham, MA).

### 4.12 Immunofluorescence staining (IF) combined with telomere FISH

Telomere‐ImmunoFISH was performed as described in (Patel & Herbig, 2017). Briefly, following the blocking step, cells were washed 3 times with PBS (5 min each) and were dehydrated by subsequent submersion in 70%, 90%, and 100% ethanol (3 min each). After dehydration, cells were left to complete air dry and were incubated with 0.5 μg/ml TelC‐Cy3 PNA probe (Panagene, Korea) in hybridization buffer (70% formamide, 12 mM Tris‐HCl pH = 8.0, 5 mM KCl, 1 mM MgCl2, 0.001% Triton X‐100, 0.25% acetylated BSA) for 5 min at 80°C, to allow denaturation of DNA and subsequent hybridization of the telomere‐specific PNA probe. The cells were then left to cool gradually and further incubated overnight in a humidified chamber at room temperature. After hybridization, cells were washed 2 times with 70% formamide/0.6X SSC (15 min each) followed by 2 washes with 2X SSC buffer (10 min each). After the last wash, immunostaining was performed as described above using anti‐53BP1 primary antibodies.

### 4.13 Image acquisition and analysis of DNA damage and Telomere dysfunction‐induced DNA damage Foci (TIF)

Images were acquired with a Zeiss Axio Observer Z1 Inverted Phase Contrast Fluorescence Microscope using a 63X oil‐immersive lens. Images were obtained using Z‐stacks and were analyzed with Zeiss ZEN 2.5 (blue edition) software. For each experiment, at least 100 cells were analyzed and the number of 53BP1 foci per cell, as well as the co‐localization of 53BP1 foci with telomeres, was assessed. Only distinct, well‐formed 53BP1 foci in each plain focus of the Z‐stack were counted for absolute number of foci per cell, as well as for colocalizations. T cells with only granular non‐focal staining pattern of 53BP1 were excluded from analysis.

### 4.14 RNA‐seq

RNA from sorted CD8+ fSA‐ßGal low and high from young and old donors (3 each) was purified using a Macherey‐Nagel RNA XS Plus kit according to the manufacturer's instructions (Macherey‐Nagel, Duren, Germany). RNA integrity was evaluated in a Bioanalyzer 2100 system, and only RNA with an integrity of number of >= 7 was used for library preparation. Libraries were constructed using the SMARTer Stranded V2 (TakaraBio) according to the manufacturer's instructions (TakaraBio, Mountain View, CA). Paired‐end sequencing was performed on an Illumina HiSeq 2500 instrument. At least 40 million reads per sample (20 million per strand) were obtained and used for downstream analyses.

### 4.15 Analysis of microarray data from Callender et al. (2018)

Publicly available Affymetrix HuGene 2.0 microarrays for N, CM, EM, and EMRA CD8+ T cells (6 each) (Callender et al., 2018) cells were analyzed using open‐source Bioconductor packages on R. All microarrays were normalized simultaneously using the robust multi‐array normalization algorithm implemented in the *oligo* package. After removing internal control probes, deciles of average expression were independently defined for each CD8+ T‐cell population. Probes falling in the lowest 4 deciles of expression were removed. Identification and removal of unidentified sources of variability were performed using the *sva* package. Probes were annotated by combining the *HuGene 2.0* and *bioMart* annotations. Hierarchical clustering and principal component analysis were used to monitor the behavior of the datasets at every step of the pre‐processing. Differential expression was performed using *limma* with the default parameters, comparing the overall expression changes of CM, EM, and EMRA relative to N CD8+ T cells. The statistical approach involves empirical Bayes‐moderated *t*‐statistics applied to all contrasts for each probe, followed by moderated *F*‐statistic to tests whether all the contrasts are zero, evaluating the significance of the expression changes observed (Ritchie et al., 2015). For significant probes, *p*‐values were corrected for multiple testing with the FDR approach using a significance level of 0.005 as a cut‐off. Probes matching these criteria that also exhibited a minimal absolute fold change of 1.4 were considered for downstream analyses.

### 4.16 RNA‐seq analysis

FASTQ files were aligned to the version 19 of the human reference genome (hg19) using *bowtie2* using the local alignment option (bowtie2 ‐‐local ‐x./hg19/hg19) which performs soft clipping of read characters to maximize alignment scores. Alignments were further processed using *samtools v1*.*9*. For genome track visualizations, alignments were normalized using *deeptools v3*.*3*.*1* using the RPGC approach to obtain 1X coverage (bamCoverage ‐b –normalizeUsing RPGC –effectiveGenomeSize 2864785220 –ignoreDuplicates –binSize 10 –verbose ‐o). Reads per exon (hg19) were quantified using the *summarizeOverlaps* package, and genes with at least 10 reads in at least 3 samples were kept. Correction of batch effects was performed with *sva*. Data transformation (regularized‐log [rld] transformation), exploratory visualization (PCA and hierarchical clustering), and differential expression analysis were performed with *DESeq2* using the default parameters as previously described (Love et al., 2014) and base R functions. Differentially expressed genes identified by this approach (absolute fold change of 1.25) were considered for downstream analyses.

### 4.17 Transcriptional co‐expression networks and functional over‐representation

For microarray and RNA‐seq datasets analyzed, the debatched normalized fluorescence and rld‐transformed counts, respectively, of the DEGs identified were used as input for the *WGCNA* (Langfelder & Horvath, 2008 *)* package and clustered using the “signed” option with default parameters with the exception of the soft‐thresholding power, which was set to 18 for microarray and 19 for RNA‐seq DEGs, respectively. The co‐expression modules identified for each dataset were visualized with heatmaps, correlation, and network plots using *WGCNA* and *pheatmap*, and functionally characterized by over‐representation tests with the *clusterProfiler* package using the Molecular Signature Database Hallmark gene sets (Liberzon et al., 2015; Subramanian et al., 2005). For the comparative analysis of microarray and RNA‐seq datasets of CD8+ T cells RNA‐seq datasets from this study with datasets from De Cecco et al. (2019) and Callender et al. (2018), the lists of DEGs derived from the *WGCNA* modules from the microarray and RNA‐seq datasets were initially characterized for intersections and disjunctive unions using *eulerr*, allowing for the identification of cell state‐ and cell type‐specific DEGs. These lists were functionally characterized with *clusterProfiler* as described above. For a quantitative visualization of the intersections and disjunctive unions of DEGs of each CD8+ T‐cell state from Callender et al., we displayed the pair‐wise correlation of the log2 fold change of DEGs common to CD8+ fSA‐ßGal high and CD8+ T cells at the CM, EM, and EMRA stages, respectively. R scripts are available upon request.

## CONFLICT OF INTEREST

The authors declare that no conflicts of interest exist.

## AUTHOR CONTRIBUTIONS

UH, PFB, RIM‐Z, and HKD conceived the study. RIM‐Z conducted experiments involving senescent human fibroblasts, RT‐qPCR, and EdU incorporations and analyzed RNA‐sequencing data. HKD collected blood, conducted FACS analysis and sorting of peripheral blood mononuclear cells and performed T‐cell proliferation assays. TV performed immunofluorescence analysis and quantification of senescence markers in sorted T cells. LGW provided cord blood and insight into neonatal physiology. UH, RIM‐Z, and HKD assembled the figures and UH and RIM‐Z wrote the manuscript with input from all authors.

## Supporting information

Supplementary Material

## Data Availability

The data that support the findings of this study are openly available in the Gene Expression Omnibus (GEO) at https://www.ncbi.nlm.nih.gov/geo/query/acc.cgi?acc=GSE169009 with reference number GSE169009.
