# Supplementary material for: Senescence‐associated β‐galactosidase reveals the abundance of senescent CD8+ T cells in aging humans
Source: Aging Cell. 2021 May 3;20(5):e13344. doi: 10.1111/acel.13344 (PMC8135084; doi:10.1111/acel.13344)
Supplement: Supplementary file 1 — Supplementary Material [file ACEL-20-e13344-s001.pdf]

Supplementary Information: Table 1 and Supplementary Figures S1-S5:

Donors	20s	60s
N	18	16
Median Age (years)	25	65
age range (years)	23-30	57-67
Male (%)	44	50
Female (%)	56	50
Caucasian (%)	61.1	62.5
African American (%)	11.1	25
Hispanic (%)	11.1	12.5
Other (%)	16.7	0

Table 1: Human subjects analyzed.

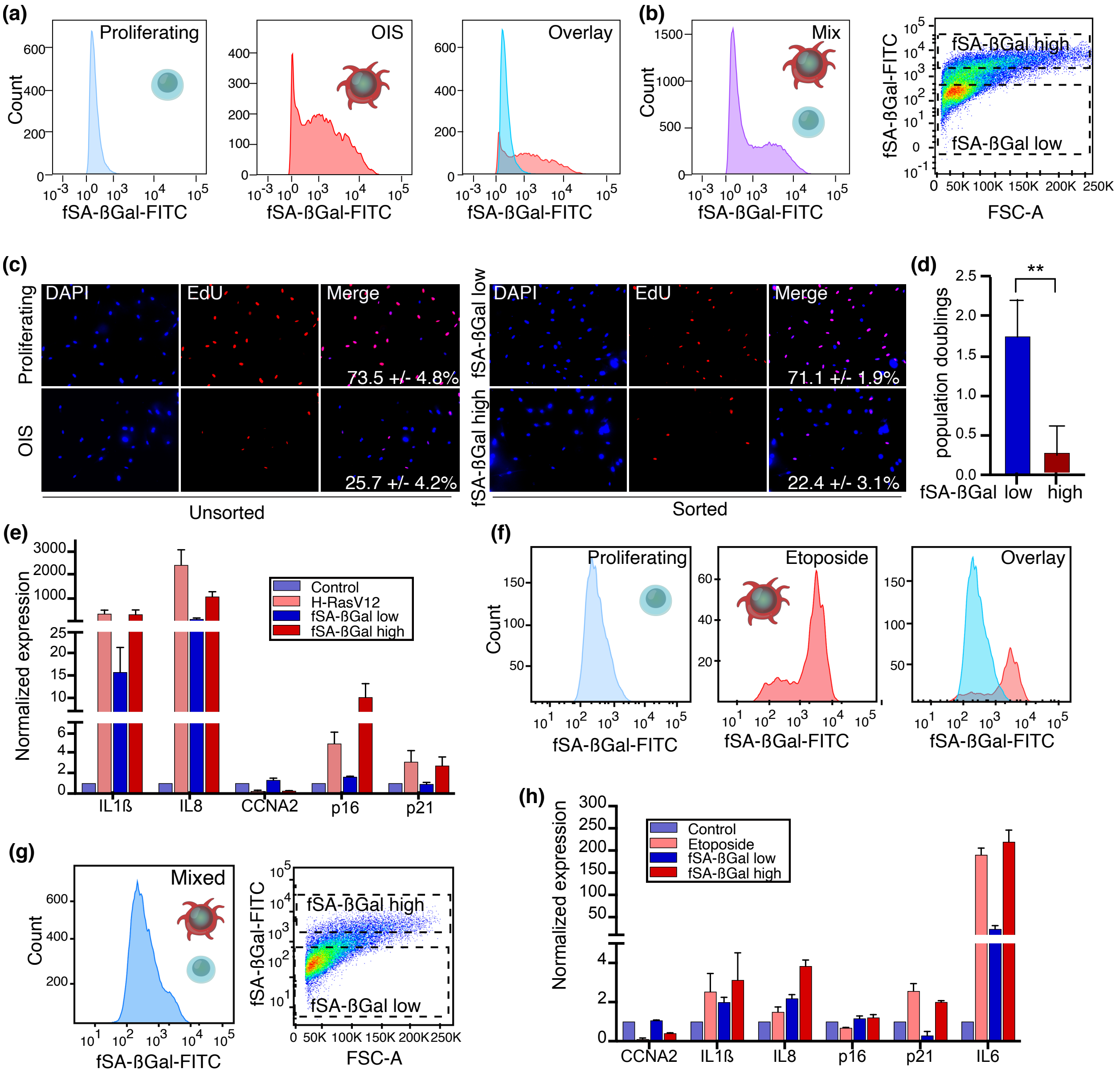

Supplementary Figure 1. Senescent human fibroblasts can be accurately isolated from mixed cell populations using a fluorogenic SA-βGal substrate. (a) Representative flow cytometry fSA-ßGal intensity profiles for proliferating and oncogene-induced senescent (OIS) fibroblasts. Overlay of the two histograms is shown on the right. (b) Proliferating fibroblasts and fibroblasts in OIS were mixed at a ratio of 3:1 and subjected to the fSA-βGal staining procedure. Histogram: fSA-ßGal intensity profile for mixed cell population; dot-plot: gating strategy to sort for fibroblasts with low and high fSA-ßGal signal intensities as indicated. (c) Immunofluorescence analysis of EdU incorporation (red) of non-sorted proliferating and OIS fibroblasts (left) and fibroblasts sorted by FACS based on gates shown in b (right), as indicated. Percentages of EdU positive cells are indicated (average and S.E.M of 2 independent experiments are shown). (d) Quantification of population doublings of fibroblasts sorted by FACS based on gates shown in b 4 days after re-plating. n = 3 +/-S.E.M. Statistical significance was calculated with an unpaired, two-tailed t-test. ** p = 0.0124. (e) Representative RT-qPCR expression profiles of indicated senescence-associated genes were determined for proliferating (control), OIS (H-RasV12), sorted, as in b, fSA-ßGal-low and fSA-ßGal-high fibroblasts.

n = 3. (f) as (a) using etoposide-induced senescent fibroblasts. (g) as (b) using etoposide-induced senescent fibroblasts. (**h)**, RT-qPCR expression profiles of indicated senescence-associated genes as in (e). n = 2.

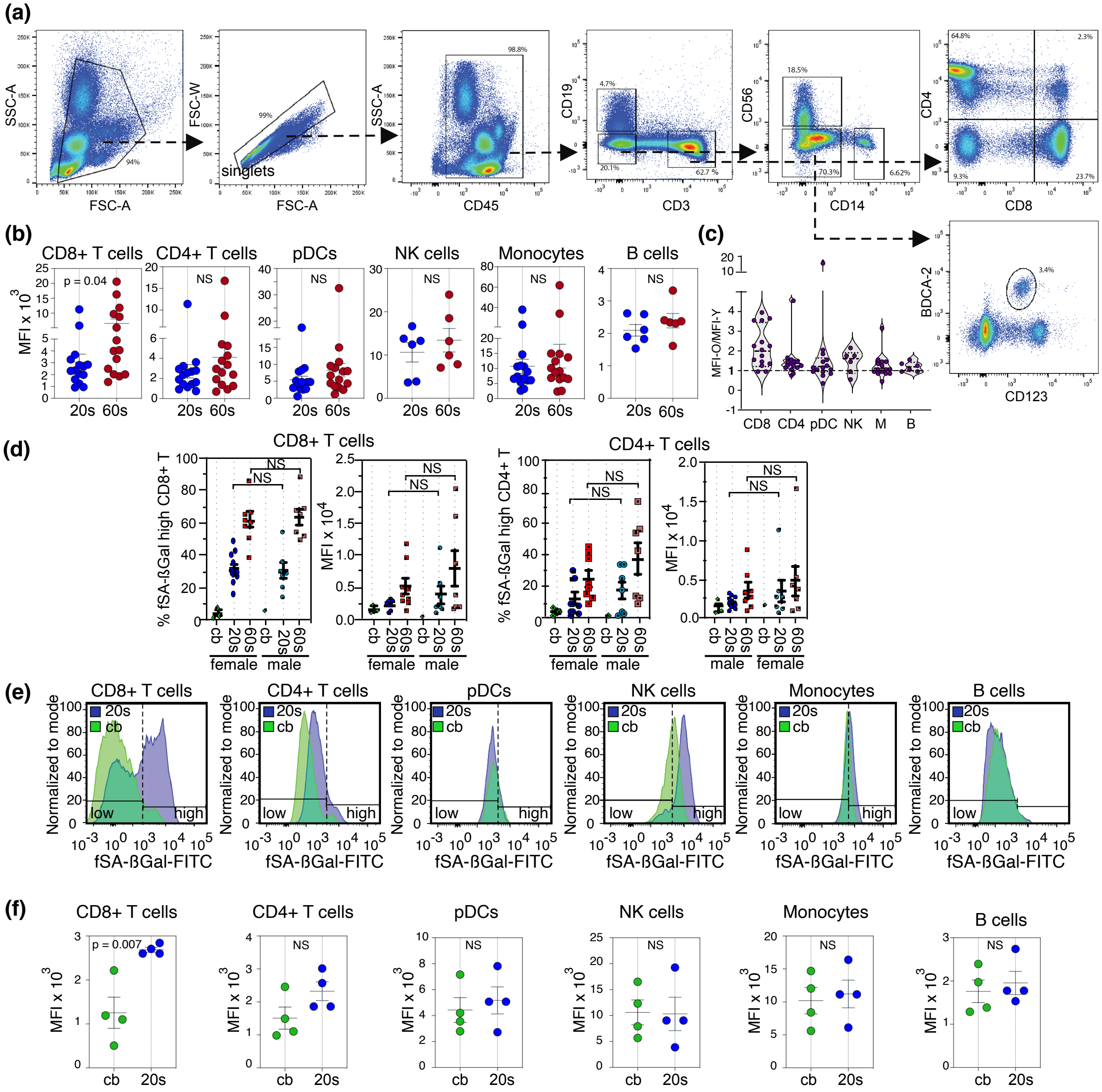

Supplementary Figure 2. fSA-βGal fluorescence signal intensities of human T lymphocytes show consistent increases with age. (a) Gating strategy to identify PBMCs, single cell events, CD45+, CD56+, CD14+, CD19+ CD4+ and CD8+ T cells, and CD123+ BDCA2+ cells. (b) Mean fluorescent fSA-βGal signal intensities (MFI; mean +/- S.E.M) of indicated PBMC subsets from young and old donors. (c) Violin plot showing ratios of MFIs between old and young donor pairs in indicated PBMC subsets. (d) Quantification of the percentages of fSA-ßGal high cells (left graph) and MFI (right graph) in cord blood (cb; green), donors in their 20s (blue) and donors in their 60s (red) for indicated PBMC subsets and stratified by sex as indicated. Data for CD8+ and CD4+ T cells are shown. (e) Representative fSA-ßGal intensity profiles and gates used to quantify fSA-ßGal high cells for indicated PBMC subsets from cord blood (green) and young (blue) donors. (f) Mean fluorescent fSA-βGal signal intensities (MFI) of indicated PBMC subsets from analyzed cord blood and young donors as indicated. Whiskers indicate mean +/- S.E.M

and are indicated for each subset. Statistical significance was determined by an unpaired, two-tailed Student's t test.*** $p<0.0001$; ** $p = 0.0005$; * $p < 0.05$; NS: not significant.

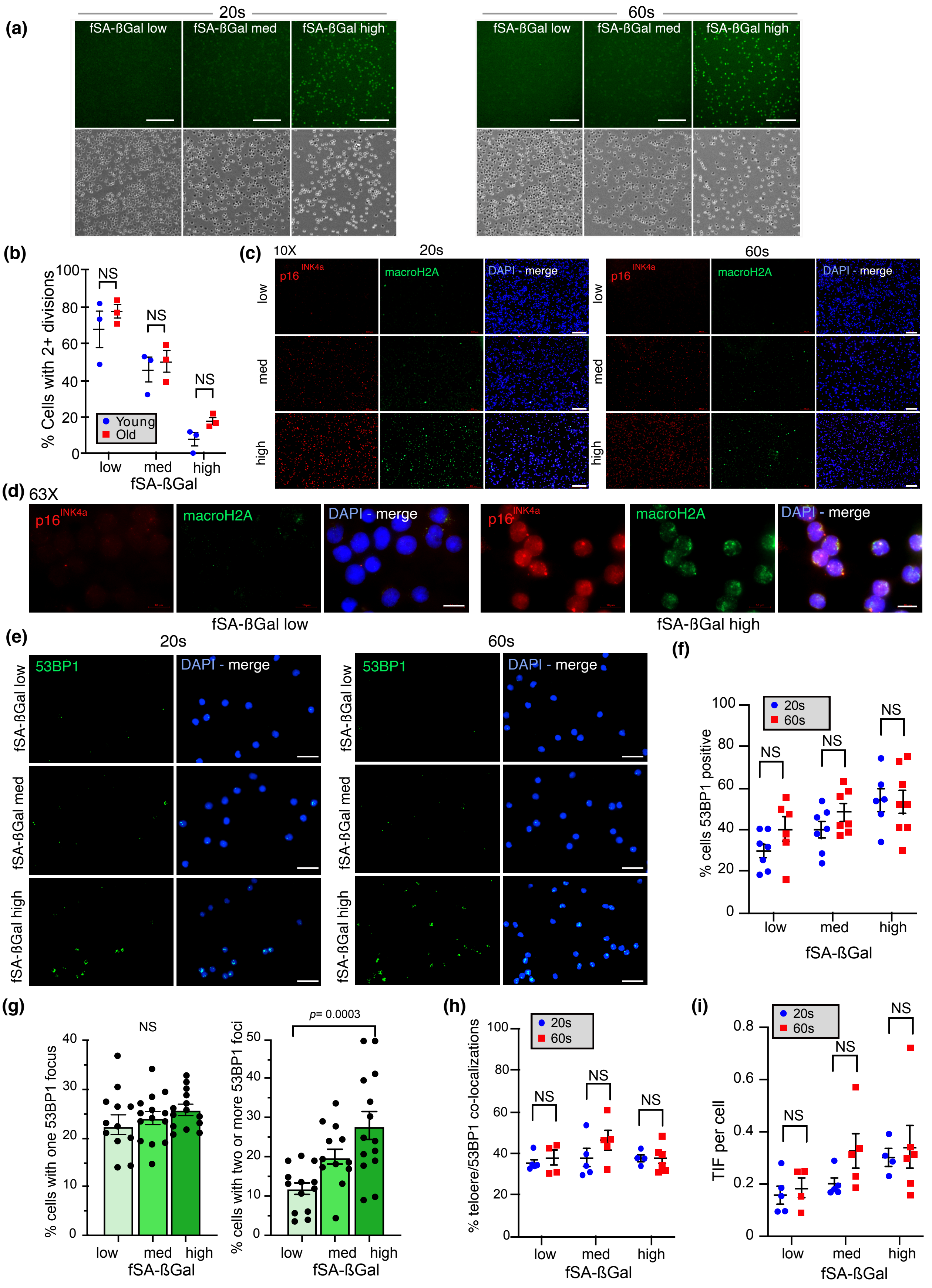

(a)
20s
60s
fSA-ßGal low
fSA-ßGal med
fSA-ßGal high
(b)
% Cells with 2+ divisions
NS
Young
Old
low
med
high
fSA-ßGal
(c)
10X
p16INK4a
macroH2A
DAPI - merge
(d) 63X
fSA-ßGal low
fSA-ßGal high
(e)
53BP1
(f)
% cells 53BP1 positive
20s
60s
(g)
% cells with one 53BP1 focus
% cells with two or more 53BP1 foci
p= 0.0003
(h)
% teloere/53BP1 co-localizations
(i)
TIF per cell

Supplementary Figure 3. CD8+ T cells with high fSA-βGal signal intensities display hallmark features of senescent cells. (a) Micrographs of CD8+ T cells that were sorted based on indicated fSA-βGal signal intensities from a donor in their 20s (left) and 60s (right). Top row (green): fSA-βGal; bottom row: phase contrast (scale bar 200 μm) (b) Quantification of more than two cell divisions of CD8+ T cells from 3 young (blue) and 3 old (red) donors following anti-CD3 and anti-CD28 stimulation for 60 h. Statistical significance was calculated with a two-way ANOVA. NS: not significant. (c) Immunofluorescence analysis of CD8+ T cells from a young and old donor sorted based on fSA-βGal signal intensities, as indicated, using antibodies against macroH2A (green) and $p16^{INK4a}$ (red). Blue: DAPI. Images were acquired at 10X magnification (scale bar 100 μm) and (d) at 63X magnification (scale bar 10 μm). (e) Immunofluorescence analysis as in B using antibodies against 53BP1 (green). Blue: DAPI. (scale bar 20 μm). (f) Quantification of CD8+ T cells positive for 53BP1 foci, sorted based on indicated fSA-βGal signal intensities from young (blue) and old (red) donors. Statistical significance was calculated with a two-way ANOVA. NS: not significant. (g) Quantification of CD8+ T cells sorted based on indicated fSA-βGal signal intensities from young and old donors combined, displaying one 53BP1 focus only (left bar graph) and two or more 53BP1 foci (right graph). Young n = 8, Old n=8. Bars and whiskers depict mean +/- S.E.M. Statistical significance was calculated with a one-way ANOVA. NS: not significant. (h) Quantification of percent 53BP1-telomere colocalizations as indicated. (i) Quantification of TIF per cell in CD8+ T cells sorted based on indicated fSA-βGal signal intensities from young (blue) and old (red) donors. Statistical significance was calculated with a two-way ANOVA (g,h). NS: not significant.

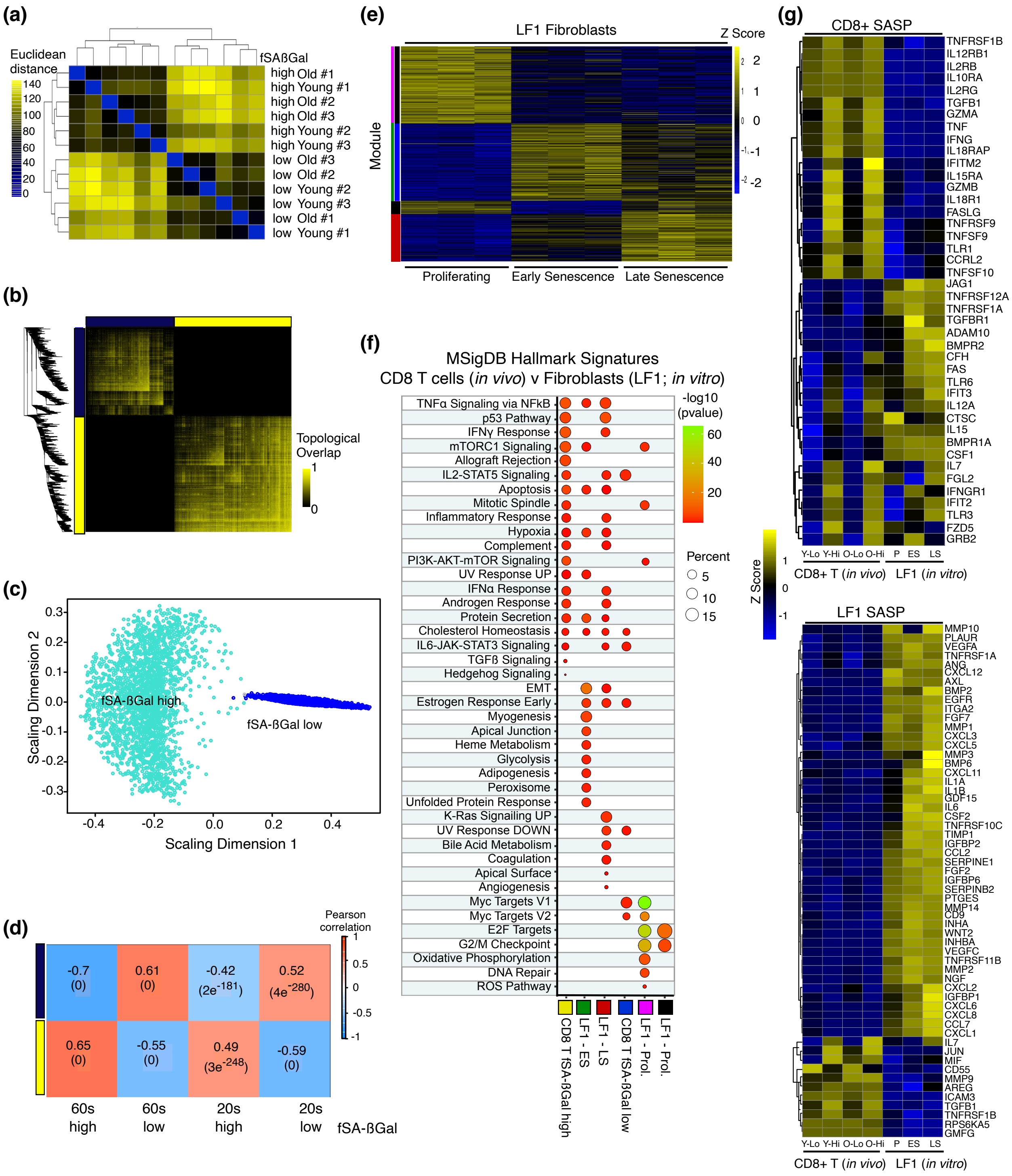

Supplementary Figure 4. Gene expression profiles of senescent human CD8+ T cells and senescent human lung fibroblasts (LF1). (a) Hierarchical clustering of r-log-regularized counts per exon (see Methods) of CD8+ fSA-ßGal-low and -high T cells from young and old donors. (b) Network plot of the 4,149 DEGs in CD8+ fSA-ßGal-high T cells. Note that two distinct modules are identified (dark blue and yellow). (c) Multidimensional scaling plot of the two gene modules (fSA-ßGal-low and -high) identified in Fig 3b. Each dot represents a single gene. (d) Correlation plot showing gene module usage in CD8+ fSA-ßGal-low and -high CD8+ T cells of young and

old donors as indicated. (e) Heatmap showing four modules of co-expressed genes specific for the indicated human LF1 fibroblast samples using an unsupervised WGCNA clustering approach. (f) Functional overrepresentation analysis comparing enrichment of MSigDB hallmark gene sets in CD8+ fSA-ßGal-low and -high T cells, as well as proliferating, early replicative senescent (ES; 4 months) and late replicative senescent (LS) human LF1 fibroblasts. Circles are color-coded according to the FDR-corrected p-value based on the hypergeometric distribution test. (g) Expression heatmaps of a selection of CD8+ T cell (top) and LF1- (bottom) specific SASP genes in low (lo) and high (hi) fSA-ßGal CD8+ T cells from young and old donors and LF1 fibroblasts in proliferation (P), early (E; 2 months) and late (L; 4 months) senescence. Each column represents the average expression of 3 independent donors (CD8+) and 3 independent experiments (LF1).

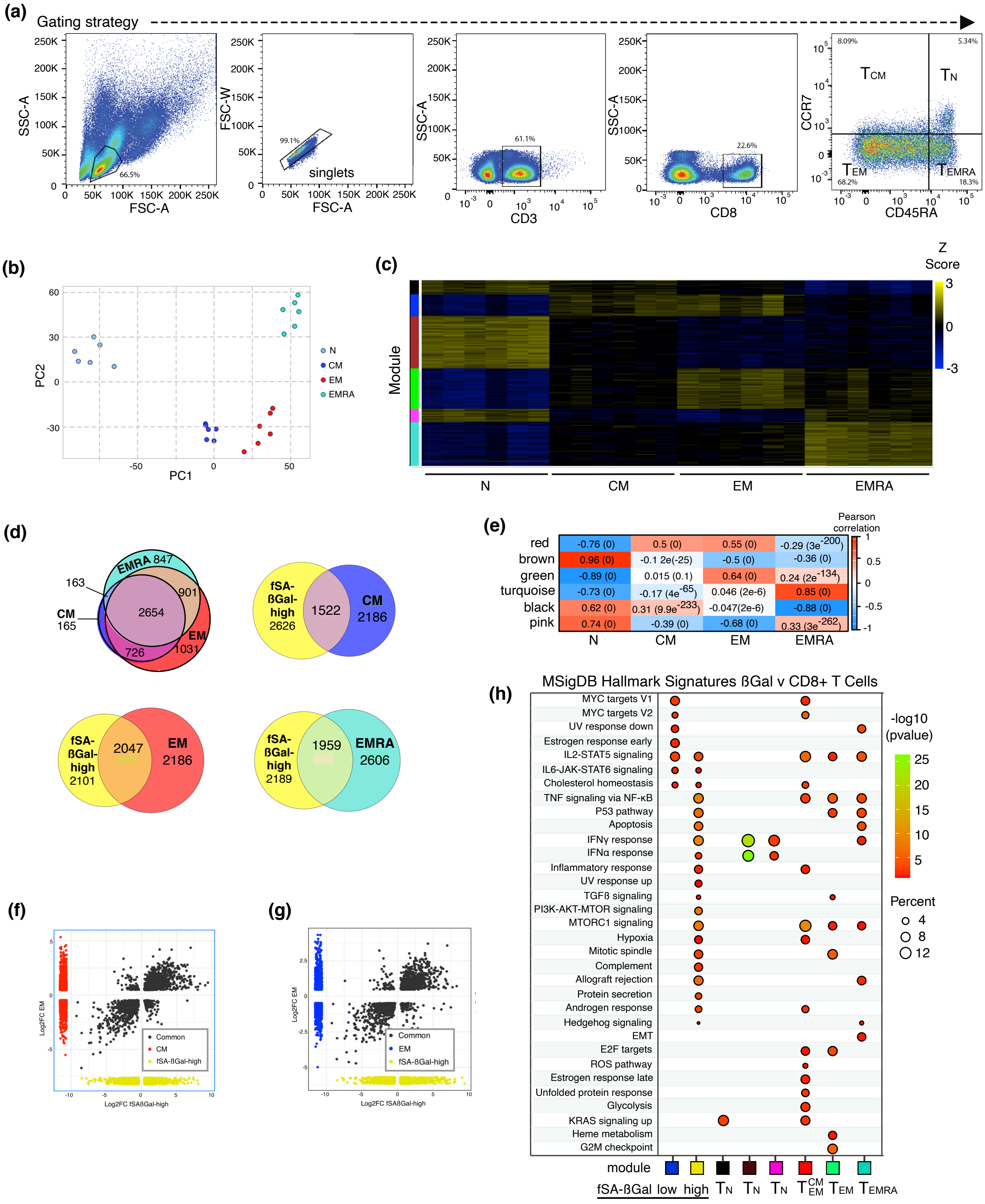

Supplementary Figure 5. Senescent CD8+ T cells are transcriptionally distinct from T_N, T_{CM}, T_{EM}, and T_{EMRA} cells. (a) Gating strategy to identify CD8+ T cell subsets by expression of CCR7 and CD45RA. (b) Principal component analysis of normalized and debatched transcriptomes of N, CM, EM and EMRA CD8+ T cells. CD8+T cell differentiation stages are color-coded.

Microarray data from Callender et al, 2018., were reanalyzed (b-g). (c) Expression heatmap of the 5,593 DEGs across each CD8+ T cell differentiation stage. Microarray data from Callender et al, 2018 were clustered using WGCNA. Each column represents an individual donor. Data are represented as Z-scores. (d) Venn diagrams portraying the intersections and disjunctive unions of DEGs in fSA-ßGal-high T_{CM}, T_{EM}, and T_{EMRA} CD8+ T cells. (e) Correlation plot showing gene module usage in T_N, T_{CM}, T_{EM}, and T_{EMRA} CD8+ T cells. (f, g) Correlation plot of the log2 fold changes of the DEGs in fSA-ßGal-high, CM (e) and EM (f) CD8+ T cells. Dark grey points represent genes expressed in both populations for each pair-wise comparison. Red dots are CM-specific genes. Blue dots are EM-specific genes. Yellow dots are fSA-ßGal-high-specific genes. (h) Functional overrepresentation analysis depicting enrichment of Molecular Signature Database gene sets in the fSA-ßGal-low and -high gene modules identified in Figure 3 and in differentiation stage-specific gene of CD8+ T cells. Note that CD8+ fSA-ßGal-high T cells enrich for most gene sets found in CD8+ T cells at every differentiation stage. Circles are color-coded according to the FDR-corrected p-value based on the hypergeometric distribution test.
